# Same-day discharge versus overnight stay after pulmonary vein isolation: an assessment on clinical outcomes and healthcare utilization

**DOI:** 10.1007/s10840-025-02077-w

**Published:** 2025-06-19

**Authors:** SR Stacey Slingerland, JLPM Maarten Van den Broek, DN Daniela Schulz, GJ Gijs van Steenbergen, LRC Lukas Dekker, AJ Alexandre Ouss, D Dennis van Veghel

**Affiliations:** 1 Department of Cardiology, Catharina Heart Centre, Catharina Hospital, P.O. Box 1350, Eindhoven, ZA 5602 The Netherlands; 2 Department of Biomedical Technology, Eindhoven University of Technology, Eindhoven, The Netherlands

**Keywords:** Pulmonary vein isolation, Same-day discharge, Overnight stay, Clinical outcomes, Quality improvement

## Abstract

**Background:**

Atrial fibrillation is increasingly prevalent and constitutes a severe economic and clinical burden. Pulmonary vein isolation (PVI) is an effective treatment. Evidence on the safety of same-day discharge (SDD) after PVI, instead of overnight stay (ONS), is limited.

**Methods and results:**

This retrospective study uses data from PVI’s performed between June 2018 and December 2020 in the Netherlands. Baseline characteristics, clinical outcome data, and healthcare utilization, extracted from two national databases, were compared between the implementation of an SDD protocol in a single center and a national benchmark where the majority is an ONS strategy. Descriptive and bivariate analyses were performed. We included data from 11,812 PVI’s,1360 in the SDD protocol group, and 10,452 for the ONS benchmark. The SDD protocol group performed 57.7% of PVI’s in SDD, while the benchmark performed 5.3% (*p* < 0.001). The SDD protocol group performed more cryoballoon PVI (90.8% vs. 39.2%, *p* < 0.001). There were no differences in bleeding (*p* = 0.830), thromboembolic (*p* = 0.893), vascular complications (*p* = 0.720), or cardiac tamponade (*p* = 0.634). Peri-procedural hospital stay was significantly shorter in the SDD protocol group (0.50 day vs. 1.52 days, *p* < 0.001), without a reallocation of health care to outpatient clinic (*p* = 0.230), emergency department (*p* = 0.132), or a higher rate of readmission (*p* = 0.092).

**Conclusion:**

The SDD protocol group with 57.7% SDD has similar complication rates and lower healthcare utilization, compared to the national ONS benchmark with 5.3% SDD, indicating that the SDD protocol is a safe and effective alternative for ONS in patients undergoing PVI. The implementation of an SDD protocol results suggests a potential reduction of nationwide healthcare utilization.

**Graphical Abstract:**

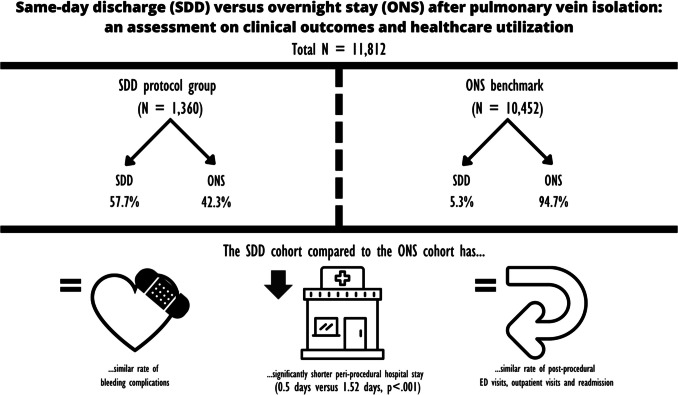

**Supplementary Information:**

The online version contains supplementary material available at 10.1007/s10840-025-02077-w.

## Introduction

Atrial fibrillation (AF) is the most common cardiac arrhythmia, associated with high healthcare costs, morbidity, and mortality [1, 2]. About 2% of the general population suffers from AF, and it is expected that this number will double or triple in the coming decades due to ageing and earlier detection [1, 2]. Catheter-based pulmonary vein isolation (PVI) is an effective first- or second-line treatment, superior to antiarrhythmic drug therapy [3–7]. PVI is the most commonly performed complex ablation procedure, but it is a resource-intense procedure and increasing in demand [8–10].

Due to advancements in PVI, procedural and clinical outcomes have seen impressive improvements, and complications have decreased significantly. The average procedure time went down from up to 6 h to slightly over 1 h [11]. Nevertheless, overnight stay (ONS) still is common practice in the majority of electrophysiology centres [12]. The combination of the sheer amount of PVI procedures and costs of in-hospital stay and limited bed availability has led the cardiology community to search for innovations to shorten the time spent in hospital. An infrastructural innovation deemed to improve the patient experience while decreasing costs is the development of same-day discharge (SDD) protocols [13–15]. Although earlier research indicated the safety and feasibility of SDD, evidence is still limited to randomized controlled trial studies or small historical cohort studies [10, 14–26]. In this study, using real-world observational data, we evaluate the clinical outcomes and healthcare utilization during hospital admission and after discharge, following the implementation of an SDD protocol in comparison to a national benchmark in which SDD is not commonly performed.

### Methods

#### Study design

This retrospective study compares the implementation of an SDD protocol in a single center (“*SDD-protocol group*”) to data from a national benchmark where the majority has an ONS strategy (“*ONS-benchmark*”) in the same period, using observational data from the Netherlands Heart Registration (NHR) and Dutch Hospital Data (DHD).

##### Inclusion and exclusion criteria

The SDD-protocol group includes patients undergoing both primary PVI and re-do PVI between June 2018 and December 2020. The ONS-benchmark comprises patients undergoing PVI (primary or re-do) from other electrophysiology centres in the Netherlands in the same period. Both groups include unique patients, counted once for either PVI or re-do PVI.

#### Pulmonary vein isolation procedure in the SDD-protocol group

In the SDD-protocol group, all patients were admitted two hours prior to the procedure and placed on a *nil per os* for 12 h. Cryoballoon (CB) ablation was the default technology for primary PVI; re-do procedures were performed with 3D mapping and radiofrequency (RF)-ablation. CB ablation was performed through unilateral venous access, using two sheaths (8 Fr and 12 Fr). Re-do procedures required bilateral venous access (two 8 Fr and one 6 Fr) and a 2 Fr arterial sheath for hemodynamic monitoring. Ultrasound guidance for venous access was not standard at the time. A specialized nurse monitored procedural analgesia and sedation. Both CB-PVI and re-do procedures with RF were performed under uninterrupted oral anticoagulation. With intravenous heparin, an active clotting time was maintained at > 300 s while dwelling in the left atrium. Protamine was administered after retracting the catheter to the right atrium, prior to the removal of the sheaths. All patients received manual compression and a 6-h compression bandage ensuring venous (and arterial) closure. A cardiac ultrasound was performed immediately after the procedure to assess pericardial effusion. The attending physician examined the groin before discharge and issued an ultrasound when deemed necessary.

#### The adoption of the SDD strategy

The SDD-protocol group is from the Catharina Hospital in Eindhoven (CHE), the Netherlands. The SDD protocol was adopted in 2018. The PVI procedure was not changed with the introduction of the SDD protocol, with the exemption of the physical examination of the groin in the evening hours, typically 1 h after removal of the pressure bandage, instead of the next day.

Procedures ending before 3 PM and free from any (suspected) complications lead to SDD by protocol; this group is called the SDD group. In case of (suspected) complications or at the operator’s discretion, the discharge could be postponed, e.g., until potential complications were resolved or ruled out. This group is called the unexpected ONS group. Patients whose procedures ended after 3 PM, stayed overnight per protocol and were discharged < 24 h. A completion before 3 PM was chosen to prevent discharge at night since the protocoled 6-h compression bandage and 1-h mobilization would otherwise lead to discharge after 10 PM. This group is the expected ONS-group. The distinction between SDD, expected ONS, and unexpected ONS is made using the SDD-protocol group’s hospital data. We are not aware of the (peri)procedural protocols of the hospitals in the ONS benchmark.

#### Endpoints per data source

##### Baseline characteristics and clinical outcomes

NHR is an independent nationwide clinical registry in which hospitals register a standard set of baseline, procedural, and outcome data for all invasive cardiac procedures [27, 28]. On an annual basis, the NHR publishes outcome data of partaking hospitals to improve the quality, safety, and transparency of care. The baseline characteristics from NHR are displayed in Table 1. The following outcome data are used: 30-day mortality, bleeding complications during admission, thromboembolic complication < 72 h, vascular complication < 30 days, cardiac tamponade < 30 days, and phrenic nerve injury (PNI) during admission. All definitions are aligned with ESC [29, 30].

**Table 1:** Baseline and procedural characteristics

	SDD protocol group (***n*** = 1150), ***n*** (%)	ONS benchmark (***n*** = 8391), ***n*** (%)	***p***-value (***χ***^2^)
**Patient**			
Sex (male)	789 (68.6%)	5,557 (66.2%)	0.108
Age (≥ 75 years)	112 (9.7%)	598 (7.1%)	0.002
BMI (≥ 25)	824 (71.7%)	5,786 (69.3%)	0.063
CHA2DS2 VASc (≥ 2)	612 (53.4%)	4,065 (49.2%)	0.002
Longstanding persistent AF	23 (2.0%)	131 (1.6%)	0.268
Prior LA ablation	123 (10.7%)	857 (10.2%)	0.613
**Procedural**			
Cryoballoon PVI	1044 (90.8%)	3288 (39.2%)	< 0.001
RF PVI	106 (9.2%)	5,083 (60.8%)	< 0.001
Additional LA ablation besides PVI	12 (1.1%)	669 (8.0%)	< 0.001

*SDD* same-day discharge, *ONS* overnight stay, *BMI* body mass index, *AF* atrial fibrillation, *LA* left atrium, *PVI* pulmonary vein isolation, *RF* radiofrequency

##### Healthcare utilization

DHD manages medical and administrative data registered by Dutch hospitals to a national registry of hospital care [31]. In this study, healthcare utilization is included as duration of peri-procedural hospital stay and healthcare utilization after discharge (emergency department (ED) and outpatient visits, in any Dutch hospital) to assess the effects of relocation or delay of care after the implementation of the SDD protocol (with a follow-up of 14 months after discharge).

#### Statistical analyses

Patients were stratified to the SDD protocol group or the ONS benchmark. Statistical significant differences between the groups were assessed for categorical variables by a chi-square test and a two-sided Student’s *t*-test for continuous variables. Results were considered statistically significant with a two-tailed *p*-value < α.05. All data analyses were performed in IBM SPSS V26.

## Results

According to the DHD data, 1360 PVIs were performed between June 2018 and December 2020 in the SDD-protocol group and 10,452 in the ONS benchmark. The SDD protocol group performed significantly more procedures in SDD than the ONS benchmark (respectively 57.7%, 5.3% *p* < 0.001). The results per year are shown in Table 1 and Fig. 1 in the Appendix, showing the flowchart of the included patients over the different cohorts.

Table 1 shows the baseline and procedural characteristics of the SDD protocol group (*N* = 1150) and the ONS benchmark cohort (*N* = 8,391), based on NHR data. Patients in the SDD protocol group are significantly older and less healthy (higher CHA_2_DS_2_VASc score) compared to the ONS benchmark. Furthermore, the SDD protocol group had more CB-PVI and fewer additional LA ablations compared to the ONS benchmark (*p* = 0.002, *p* = 0.002, *p* < 0.001, *p* < 0.001 respectively). 

Table 2, using both DHD and NHR data, shows mortality, complications, and healthcare utilization after PVI. There are no differences in mortality or complications except for PNI; the ONS benchmark has fewer events (0.6%) compared to the SSD protocol group (1.3%) (*p* = 0.005).

**Table 2:** Mortality, complications, and healthcare utilization

	SDD protocol group	ONS benchmark	*p*-value
***Clinical outcomes***	***N* = 1360**	***N* = 10,452**	
30-days mortality (*n*, %)	0 (0.0)	0 (0.0)	n/a
Bleeding complication during hospital stay (*n*, %)	6 (0.5)	42 (0.5)	0.830
Thromboembolic complication (< 72 h) (*n*, %)	2 (0.2)	17 (0.2)	0.893
Vascular complication (< 30 days) (*n*, %)	14 (1.2)	119 (1.5)	0.720
Cardiac tamponade (< 30 days) (*n*, %)	5 (0.4)	48 (0.6)	0.634
Phrenic nerve injury during admission (*n*, %)	15 (1.3)	49 (0.6)	0.005
***Healthcare utilization***	***N* = 1150**	***N* = 8391**	
Peri-procedural hospital stay (days), (mean, SD)	0.50 (1.13)	1.52 (2.51)	< 0.001
ED visit within 7 days after PVI (*n*, %)	52 (3.82)	495 (4.73)	0.132
Outpatient visits within 14 months, average visits (mean, SD)	2.61 (2.34)	2.74 (2.72)	0.092
Readmission within 4 months (*n*, %)	214 (15.74)	1780 (17.03)	0.230

*SDD* same-day discharge, *ONS* overnight stay, *ED* emergency department

The mean duration of hospital admission was significantly shorter in the SDD protocol group (0.50 days), compared to the benchmark (1.52 days) (*p* < 0.001). There is no statistical difference in healthcare utilization in terms of ED visits < 7 days after PVI, readmission < 4 months, or post-PVI outpatient visits < 14 months.

Last, Table 2 in the Appendix presents additional analyses comparing SDD and (expected and unexpected) ONS cohorts within the SDD protocol group. It displays the effectuation of the SDD protocol and differences in baseline and procedural characteristics and clinical outcomes. In the single center that implemented the SDD protocol, SDD was effectuated in 82.8% of cases. Compared to the unexpected ONS subgroup, patients in the expected ONS subgroup were statistically significantly more often male (*p* = 0.020), more frequently underwent procedures using CB technique (*p* < 0.001), and more often received additional LA ablation besides PVI (*p* = 0.026). There were no statistically significant differences in mortality or clinical outcomes, with the exception of phrenic nerve injury during admission, which occurred more frequently in the unexpected ONS subgroup (*p* = 0.007).

## Discussion

This retrospective study, based on real-world data, compared clinical outcomes and healthcare utilization between a single center SDD protocol group and a national benchmark that relies mostly on an ONS strategy. Findings revealed no differences in mortality, bleeding complications, or post-procedural healthcare utilization. Remarkably, these outcomes were consistent despite the SDD protocol group comprising significantly older patients with a higher CHA2DS2 VASc score compared to the ONS benchmark. Furthermore, although peri-procedural hospital stay in the SDD protocol group was significantly shorter than in the benchmark, this did not lead to a reallocation of care, evidenced by similar rates of hospital readmission, ED visits, and outpatient visits. These results further contribute to the notion that the implementation of an SDD protocol in this subpopulation is a safe and more efficient alternative to ONS with lower healthcare utilization.

The SD protocol group included a statistically significantly higher proportion of CB-PVI procedures compared to the ONS benchmark, 90.8% versus 39.2%, and hence had a statistically significantly higher percentage of PNI compared to the ONS benchmark. PNI is a well-documented complication of CB-PVI, with previous studies indicating that CB-PVI is associated with up to ten times greater odds of PNI compared to RF-PVI [32–34]. Importantly, PNI is often reversible, and this study showed no differences in other clinical outcomes between the groups. This aligns with a recent meta-analysis by Huang et al., which demonstrated similar efficacy and safety between CB-PVI and RF-PVI [35]. Moreover, despite differences in ablation methods, no difference in healthcare utilization was observed, indicating that PVI can be safely performed in SDD.

A common cause of concern when implementing an SDD protocol is the possible increase of complications occurring after discharge and/or the reallocation of healthcare utilization, such as more ED visits [36]. However, our study, in accordance with recent studies, disputes this notion, showing no increase, substitution, or relocation of healthcare utilization, nor is there an increase in vascular complications [26, 36].

Not all patients who were scheduled for SDD are discharged the same day, hence the unexpected ONS group. Within the single center that implemented a SDD protocol, the planned SDD was effectuated in 82.8% (Table 2, appendix), which is similar to results from other studies where planned SDD was effectuated in 79% to 99% of the cases [10, 14–26]. In the two studies with the highest percentage of effectuated planned SDD, recovery after PVI (including duration of groin compression bandage) was 3–4 h as opposed to the 7 h in the SDD protocol group in this study [10, 26]. As expected, complications, such as PNI, occurred more often in the unexpected ONS group, compared to the SDD group. This complication is most often noted during the procedure, leading to a switch from planned SDD to unexpected ONS by protocol. Similarly, cardiac tamponade is another complication that is typically identified during the procedure and can also result in a switch from planned SDD to unexpected ONS. A slight 1% difference in cardiac tamponade is noted when comparing SDD versus ONS cohorts in general, which occurred more often in the ONS group compared to the SDD group. This can also be attributed to the primary use of CB-PVI in the SDD group, as CB-PVI is associated with a lower incidence of cardiac tamponade [37, 38]. Noteworthy, there were no statistically significant differences in baseline characteristics between the unexpected and expected ONS subgroups, aside from sex. This suggests that peri-procedural complications, rather than baseline characteristics, were the primary drivers for deviation from the planned SDD pathway.

Lastly, the SDD protocol was adopted to increase patient satisfaction, patient value, and efficiency. The implementation of the SDD protocol has the potential to decrease healthcare costs since it prevents costs related to ONS such as the usage of hospital beds during the night, night nurses, and costs related to hospital admission [39]. In times of scarce resources and rising costs, SDD after PVI may be considered a safe and efficient approach to provide health care for this group of patients, as also supported by the previously mentioned literature studies. Our current findings support the feasibility of this approach; hence, we suggest the implementation of an SDD protocol. The 94.7% of ONS in the benchmark suggests a potential opportunity to reduce nationwide healthcare utilization.

### Strengths, limitations, and recommendations for future research

Several studies have demonstrated the safety, feasibility, and cost-effectiveness of SDD-protocols [10, 14–26]. However, these studies often use historical cohorts, afternoon procedures, or a single center as the control group. This study’s strength lies in the completeness and high quality of the data from NHR and DHD, minimizing potential confounders such as the operator learning curve and providing a larger control group compared to only using afternoon procedures. The national scope of the data ensures a representative view of the Dutch AF population undergoing PVI. The usage of DHD data allows complete follow-up information on all patients, including those from hospitals other than the one performing the PVI, reducing the risk of underestimating post-procedural events.

The differences in procedures and processes are an important limitation. The SDD protocol group has a significantly higher percentage of CB-PVI compared to the benchmark, which could act as a confounder in this study. CB-PVI requires a larger diameter venous access sheath with a potentially increased risk of access site complications [23, 40]. Except for PNI, RF and CB-PVI have been demonstrated to have an equal safety profile; hence, we expect the effect of the energy source on post-discharge safety to be negligible [38]. Furthermore, analysis of important procedural characteristics, such as protamine use, monitoring of activated clotting time, and uni- or bi-lateral access, was not feasible, limiting our ability to correct for these potential confounders.

Another limitation is the number of inclusions from NHR is slightly lower than the number of DHD inclusions due to differences in registration; the NHR utilizes “PVI” as the inclusion criterion while in DHD the broader term “left atrium catheter ablation’”is used, thereby including, e.g., left atrial tachycardias. However, these differences are minimal and their impact on the analyses is expected to be negligible. Second, DHD data were only provided at a group level, preventing correction for potential confounding factors. Nonetheless, bivariate analyses revealed minimal differences in patient and procedural characteristics, indicating the SDD protocol group produced similar outcomes while treating an older population with higher stroke risk.

Another restraint of this study is that the extrapolation of our results might be limited. The SDD protocol group is based in a high-volume, tertiary heart center; the extensibility of these results towards low-volume and less experienced centers is uncertain, and applying them to centers predominantly performing RF or pulsed field ablation (PFA) procedures is challenging. In addition, given possible differences in socioeconomic status, healthcare access, and patient health status, the AF population in the Netherlands may not be fully representative of other countries, potentially limiting the generalisability of our findings.

For future research, it is recommended to add a costs perspective to assess the total impact of the implementation of an SDD protocol in terms of healthcare costs, especially in the context of rising healthcare costs, scarce resources, and the need for efficient utilization. Furthermore, it is recommended to assess whether comparable outcomes can be achieved across a broader range of hospital settings, including lower volume centers with varying levels of experience.

## Conclusion

This study assessed differences in clinical outcomes and healthcare utilization following PVI after the implementation of an SDD protocol in a tertiary referral center compared to a national benchmark that largely relies on an ONS strategy. The SDD protocol allowed for overnight admission when clinically indicated. Results demonstrate no difference in mortality and complications, except regarding PNI due to a higher percentage of CB-PVI. Peri-procedural hospital stay in the SDD protocol group was significantly shorter than in the ONS benchmark, without leading to a reallocation of healthcare to the ED department or outpatient clinic, resulting in a decrease of healthcare utilization. These results contribute to the notion that SDD in this subpopulation is a safe and effective alternative to ONS for patients undergoing PVI. Last, the percentage of ONS in the benchmark suggests a potential opportunity to reduce nationwide healthcare utilization.

## Supplementary Information

Below is the link to the electronic supplementary material.

Supplementary file1 (DOCX 39.9 KB)

## Data Availability

The data underlying this article were provided by the Netherlands Heart Registration (NHR) and Dutch Hospital Data (DHD) by permission. A request can be submitted to NHR and DHD to access these data.
